# Annotations

**Published:** 1891-10-24

**Authors:** 


### Annotations.

**The Practical Man.**

There is nothing one likes better to see than a boastful vapourer hoist with his own petard. It has long been the fashion among young men in laboratories to sneer at what they describe as "common sense," and to make gibes and flouts and jeers at what they derisively call the "practical man." But now that Mrs. Besant and Colonel Olcott, with their cloudy hosts of Mahatmas, are claiming to rank as scientists, and to deride the practical man and the man of common sense, it will be a little amusing to observe what the young lions of the laboratory will think it desirable to do. "R. W. S.," writing to the *Times* a few days ago on the subject of "Dreamers," makes some remarks which are very pertinent to the whole question, and which those who love to think of themselves as "scientists emancipated from practice" will do well to take a little to heart. "Of dreamers," says "R. W. S.," "the world is in need, and of dreamers the world has now and has always had abundance. What the world wants, and what the world is and always has been insufficiently supplied with, is dreamers who are also, both by nature and by training, what is meant by the term 'practical.' It is these dreamers, and these dreamers alone (that is, the 'practical' dreamers) who are not liable to the imputation justly attributed to a well-understood category or class, that their labours are branded with the curse of absolute sterility." This is a word of pure gold. As vanity in the pulpit and as frivolity at the graveside, so are pedantry and affected superiority in the scientific laboratory. Many men, more particularly young men, who delude themselves with the idea that they are men of science, affect to hold, as we have said, the mere practical man in derision. The plain, simple, and demonstrable truth is that the man who is not practical is not scientific. If science boasts of one thing more than of another it is that she deals with "things," not merely with "words." How can a man deal with things all the day and every day of his life without being practical? He must be practical if he deals with "things," even though he does not know it; and if he is practical and does not know it, what is he but a vain fool? Even the mathematician, who deals with figures and mere abstract calculations, becomes in the long run the most severely practical of men—too practical, indeed, in many cases for his own and other people's pleasure. Most divorces result in sterility; but there is no divorce which is more absolutely and hopelessly sterile than that between science and practice.

**A Pillar of Fortune.**

A writer in the *Globe*, last week, described three pillars on which, he said, Jay Gould's colossal fortune of thirty millions sterling had been erected. One of those pillars was affirmed to be "A complete emancipation from moral fastidiousness." The present writer is not a financier; but as he travelled to Tunbridge Wells the other evening a City acquaintance sitting opposite suddenly gave way to a quiet but very amused laugh, and then read out the sentence—"a complete emancipation from moral fastidiousness." As a City man accustomed to the intricacies of finance, he thought the expression one of the cleverest and most amusing he had ever met with. It undoubtedly is amusing. What the writer probably meant to say was that a particular person's fortune had been erected on trickery of all sorts, as one of its main pillars. But the idea of morality being a kind of slavery and imprisonment, from which emancipation and freedom are to be sought, is distinctly modern and clever. No doubt many people, the majority perhaps, in this country will be shocked at the state of things hereby partly disclosed. The question is—ought they to be shocked? Of course we all know that religion will justify them in being shocked, and will denounce the "complete emancipation from moral fastidiousness" as grossly immoral and wicked. But many people are not satisfied with religion as a basis of morals. Such people would probably like to hear what science has to say on the subject. Science settles the business at once. If scientific experiments were conducted, and scientific results were announced by persons who pride themselves on their complete emancipation from moral fastidiousness, what would be the consequence? It would be this and nothing less—the complete destruction of science. Pure science is pure honesty, pure candour, pure truth; these and nothing else. The scientist in his laboratory, or out in the world observing the midnight stars, tells exactly what he sees, and neither adds, subtracts, nor modifies. If he were to begin to draw upon his imagination for the purpose of enhancing his scientific fame, as Jay Gould is said to have enhanced his fortune by emancipation from moral fastidiousness, he would be ruined at once as a man of science, and ruined for ever. No scientific man would ever trust him any more; and that want of confidence would be the signing of his scientific death warrant. It is curious and instructive that as popular religion becomes more lax science becomes more stern. Religion is giving up the bottomless pit: science will have to rediscover it. There is that in the nature of the true man of science which not only insists on truth but hates lies with a stern and undying hatred; which not only insists upon absolute candour and honesty, but would consign cheats, if not to eternal flames, at least to flames which would burn out and destroy their knavery. It is not always perceived, but it is undoubtedly the fact that science, though a comparatively silent, is yet one of the noblest of witness bearers for truth, purity, and simplicity of all kinds. Jay Gould, or any other man who has emancipated himself from moral fastidiousness, is the sort of person who would have won indelible disgrace and not honour if he had carried on his activities in science instead of in finance.

**"I go on for Ever."**

For the one hundred and seventy-third year in succession the Harveian Oration has just been delivered, and Dr. W. H. Dickinson was the lecturer. Is it possible that Harvey has now at last been sufficiently admired and praised? It was his good fortune to bring to demonstration theories which had been floating about in medical minds for hundreds of years, and for the excellent work then done posterity owes him a deep debt of gratitude. But is it the best or the only way of showing our gratitude that we should set up a venerable gentleman every succeeding year, to tell us for the hundred and seventy-second or the hundred and seventy-third time the exact date of Harvey's birth; the coincidence of the destruction of the Spanish Armada with his tenth year; the fact that Elizabeth was on the throne when he was born, and that Cromwell had cut off King Charles' head before he died; that one of his contemporaries was Shakespeare, and that Shakespeare was a great dramatist; that another was Milton, and that he was a great poet; that a third was Bacon, and that he was a great philosopher; and that a fourth was Newton, and that he immortalised an apple, the law of gravitation, and himself? Is it worth while to keep on telling us, moreover, that Harvey had much prejudice to overcome in the obstinate minds of his medical contemporaries? If the lecturer were to leave the purely academical part of his business and to make a vigorous onslaught upon the prejudices of his own medical contemporaries, one might consent to read with patience the academic preface which brought him to such a practical result. But what Harveian orator ever yet ventured to hint that there was any prejudice in the medical mind of his audience? We do not say that the Harvey lecture should be disestablished, or that the orator should be disendowed. Disendowment has no charms for us. But surely a better use might be made of the Harveian endowment by the chosen lecturers than the repetition, year after year, of the same unmitigated platitudes. Harvey was a distinguished man—let it be admitted once for all; and let the lecturers who stand under the shadow of his name give some proof in their lectures that they have at least striven to work as he worked, to fight prejudice as he fought it, and to earn immortal fame by doing something, however little, to advance the useful knowledge and the practical progress of mankind.

